# Impact of psychological problems in chemical warfare survivors with severe ophthalmologic complication, a cross sectional study

**DOI:** 10.1186/1477-7525-10-36

**Published:** 2012-04-12

**Authors:** Gholamhosein Ghaedi, Hassan Ghasemi, Batool Mousavi, Mohammad Reza Soroush, Parvin Rahnama, Farhad Jafari, Siamak Afshin-Majd, Maryam Sadeghi Naeeni, Mohammad Mehdi Naghizadeh

**Affiliations:** 1 Assistant professor of psychiatry, Shahed University, Tehran, Iran; 2 Associate professor of ophthalmology, Shahed University, Tehran, Iran; 3 Prevention department, Janbazan Medical and Engineering Research Center (JMERC), Tehran, Iran; 4 MD. Janbazan Medical and Engineering Research Center (JMERC), Tehran, Iran; 5 Assistant professor, department of midwifery, faculty of nursing and midwifery, Shahed University, Tehran, Iran; 6 Assistant professor of social medicine, Shahed University, Tehran, Iran; 7 Associate professor of neurology, Shahed University, Tehran, Iran; 8 General practitioners, MD, Shahed University, Tehran, Iran; 9 Department of Community Medicine, Fasa University of Medical Sciences, Fasa, Iran; 10 Department of ophthalmology, Shahed University School of Medicine, Tehran, Iran, P.O Box: 14155-7435

**Keywords:** Sulfur mustard, Psychological status, Ocular complication, War veterans

## Abstract

**Background:**

Sulfur mustard (SM) has been used as a chemical warfare agent since the early twentieth century. Despite the large number of studies that have investigated SM induced ocular injuries, few of those studies have also focused on the psychological health status of victims. This study has evaluated the most prominent influences on the psychological health status of patients with severe SM induced ocular injuries.

**Methods:**

This descriptive study was conducted on 149 Iranian war veterans with severe SM induced eye injuries. The psychological health status of all patients was assessed using the Iranian standardized Symptom Check List 90-Revised (SCL90-R) questionnaire. The results of patients' Global Severity Index (GSI) were compared with the optimal cut-off point of 0.4 that has previously been calculated for GSI in Iranian community. The Mann-Whitney U test, T tests and effect sizes (using Cohen's d) were employed as statistical methods. Data were analyzed using SPSS software.

**Results:**

The mean age of patients was 44.86 (SD = 8.7) and mean duration of disease was 21.58 (SD = 1.20) years. Rate of exposure was once in 99 (66.4%) cases. The mean GSI (1.46) of the study group was higher compared to standardized cut off point (0.4) of the Iranian community. The results of this study showed that the mean of total GSI score was higher in participants with lower educational levels (effect size = 0.507), unemployment (effect size = 0.464) and having more than 3 children (effect size = 0.62). Among the participants, 87 (58.4%) cases had a positive psychological history for hospitalization or receiving outpatient cares previously and 62 (41.6%) cases had a negative psychological history. In addition, the mean of GSI in participants with negative psychological history was lower than those with positive psychological history (Mean Change Difference = -0.621 with SD = 0.120). There was a significant difference between positive and negative psychological history with respect to GSI (P < 0.001).

**Conclusion:**

The study showed that severe ophthalmologic complications in chemical survivors are accompanied with destructive effects on psychological health status. Appropriate management may improve psychological health status in these patients.

## Background

Since the early twentieth century, sulfur mustard (SM) (2, 2'-dichlorodiethyl sulfide, HD) has been used as a chemical warfare agent. The use of chemical weapons in the conflicts around the world is a breach of international law and a serious violation of human rights [1]. More than 100,000 Iranians have been injured by SM and majorities are still suffering from long term complications of exposure. Severe long-term effects on various organs may appear or continue for decades after exposure [2].

SM may induce many chronic and delayed destructive lesions in the ocular surface and cornea, leading to progressive visual deterioration and ocular irritation [3]. Chronic and long term ocular discomfort and the constant fear from impending blindness induce a continuous and long lasting distress that may cause different types of psychological disorders [4]. The psychological sequela in military veterans may persist as long as 50 years after exposure to mustard gas [5]. Psychological symptoms including depression, anxiety, somatization, behavioral disorders (phobia and fear from closed spaces), decreased sexual affinity, aggressiveness, sleep disorders, and early tiredness were more common in SM exposed patients [6,7].

As great stressors, wars have a major effect on psychological health status and overall quality of life. In a survey that has reviewed 60 studies, mean psychological disorders in a normal population were 3.6% before the World War Ι, but reached up to 20% after the war [8]. In 72 wars, the overall General Health Questionnaire [GHQ] scores of injured men were significantly changed, suggesting relative psychological vulnerability [9]. Veterans with full PTSD reported reduced physical health, higher rates of chronic illness and disability, greater functional impairment, and higher likelihood of health care supports [10]. In a large sample of Gulf War veterans with verifiable exposure to nerve or mustard gas, female, nonwhite, and older individuals were more likely to have a mental disorder and reported poorer current health status [11]. These situations warrant long term and expensive psychosocial supports [12]. Blanchard et al. also showed that veterans of the first gulf war (GW1) had a higher prevalence of psychiatric disorders [13]. With respect of quality of life, the St. George Respiratory Questionnaire showed poor quality of life in patients with chronic obstructive lung disease induced by chemical warfare [14]. PTSD can also affect quality of life, impairing psychosocial and occupational functioning as well as overall well-being [15]. PTSD can appear 10-20 years after a primary war-related trauma but may be overlooked or ignored. Somatic symptoms can develop along with PTSD into a seriously complicated condition that requires skilled management [16].

Psychological status could be assessed by a variety of tests. In this study we use the Symptom Check List 90 (SCL-90-R) [17,18].

The quality of life in this group of patients has previously been evaluated by Mousavi et al. and the presence of a current psychological problem has reported in 32.9% of all patients [19]. Indeed, these two studies are part of a national needs assessment project for war survivors. In the present study, the SCL-90-R was used to evaluate all 9 psychological dimensions and the global severity index (GSI) of the same group of patients. The results of this study may help to identify social or individual factors that are effective on psychological health status of patients suffering from severe eye injuries induced by chemical agents. In doing so, the present paper reports both somatic and psychosomatic symptoms experienced by war survivors, which has been somewhat neglected in the current medical literature.

## Methods

### Study design and participants

This descriptive cross sectional study was conducted on 149 participants with eye injuries due to SM exposure. The exposures were confirmed based on the documented previous military history and medical records of participants. Based on the chart of the Iranian Ophthalmic Foundation of Martyrs and Veterans Affairs [20], those veterans who were categorized as severe SM induced ocular involvement were invited to participate in this study from all provinces of Iran. Common ocular findings in this group include corneal ischemia, vascular abnormalities, neovascularization, melting, thinning, hyaline deposition, or diffuse corneal opacity [21]. The study methodology was approved by the Ethics Committee of the Janbazan Medical and Engineering Research Center (JMERC) and Shahed University, Tehran, Iran. Written informed consent was provided to all participants before the study, or the participants were otherwise excluded.

### Patients' evaluations

Demographic characteristics, SM and wartime exposure, and psychological health status data were collected from the participants. The demographic information included age, level of education, marital status, employment status, number of the children, and frequency of exposure to SM. Additional war related injuries or co-morbidity and a history of any psychological visit/treatment were also recorded. All participants were examined by both an ophthalmologist and a psychologist. All data were recorded in separate professional forms. The SCL-90-R questionnaire [22] was used in this study and the psychological health status of all patients was evaluated by three psychologists with identical training. The SCL90-R questionnaire includes 90 questions on 9 different psychological dimensions: 1) somatization, 2) obsessive-compulsive, 3) interpersonal sensitivity, 4) depression, 5) anxiety, 6) anger-hostility, 7) phobic anxiety, 8) paranoid ideation, and 9) psychoticism. The severities of psychological discomfort were graded as normal, mild, moderate, severe, or very severe [23]. The psychological interviews took about 30-45 minutes in a private environment.

### Principles and values

SCL90-R test has previously been standardized for Iran with acceptable validity and reliability [24,25]. GSI score was used for evaluation of psychological health status in this study. GSI score was calculated using the sums of the nine symptom dimensions plus the seven additional items not included in any of the dimension scores, and dividing those sums by the total number of items to which the individual responded. Those participants who had previously received psychological cares (hospitalization/outpatients) were considered as positive psychological history, otherwise considered as negative psychological history. The mean score of Positive Symptom Total (PST) and Positive Symptom Distress Index (PSDI) were used to compare the participants with negative and positive psychological history. The PST is a count of all the items with non-zero responses and reveals the number of symptoms that the respondent reports experiencing. The PSDI is the sum of the values of the items receiving non-zero responses divided by the PST. This index provides information about the average level of distress the respondent experiences [22]. An optimal cut-off point" of 0.4 was considered based on the standardized test results for Iran [24,25].

### Data Analysis

Descriptive analyses were carried out to explore the data. To determine the relationships between dependent and independent variables, Mann-Whitney U test, and T test were performed. The effect sizes of each individual item were calculated based on the Cohen's d test. Cohen's d is an effect size index used in conjunction with other statistical tests such as T test to determine the standardized difference between the two means concerning the magnitude of sample size [26]. Analysis of all data was performed using the SPSS software and a P ≤ 0.05 was considered significant.

## Results

### Demographic information

149 patients were included in this study. The mean age of the patients at the time of study was 44.86 (SD ± 8.7) years and the ages ranged from 21 to 75 years. The mean age of the patients at the time of injury was 23.32 (SD ± 8.5) years. 69 (46.3%) had attained primary or secondary levels and the 80 (53.7%) had acquired higher education. The majority of participants were married (99.3%). Reproductive history showed that all married survivors had children. More than half of survivors 90 (60.4%) were unemployed. More than two-thirds (66.4%) of the survivors had only one contact history to mustard gas during the war. Co-morbidity was reported in 74 cases (49.7%). A positive psychological history of hospitalization or outpatient cares were recorded in 87 (58.4%) and a negative history in 62 (41.6%) of the cases. Demographic characteristics of the participants are demonstrated in Table 1.

**Table 1:** Demographic characteristics of the study sample (n = 149)

Demographic items	Status	Frequency	%
**Duration of education(years)**	< 12	69	46.3
	
	≥ 12	80	53.7

**Marriage status**	Married	148	99.3
	
	Unmarried	1	0.7

**Alive child**	1-2	50	33.8
	
	3-5	73	49.3
	
	> 6	25	16.9

**Employment status**	Employed	59	39.6
	
	Unemployed	90	60.4

**Number of exposure**	Once	99	66.4
	
	Twice	36	24.2
	
	More	14	9.4

**Psychological history**	Positive	87	58.4
	
	Negative	62	41.6

**Co-morbidity**	Yes	74	49.7
	
	No	75	50.3

### Exposure parameters

In general, most chemical warfare survivors had at least 3 to 5 symptoms related to SM exposure. The mean percentage of general severity index based on data bank of the Veterans and Martyrs Affairs Foundation was 58.85% (SD = 14.8). Mean duration of SM exposure was 21.58 years (SD = 1.2).

### Psychological dimensions

The mean of GSI in survivors of chemical warfare with ophthalmologic complications was 1.46 (SD = 0.72), Higher mean scores were present in the somatization 1.98 (SD = 0.84), obsessive-compulsive disorder 1.51 (SD = 0.85), anxiety 1.56 (SD = 0.86), and depression 1.51 (SD = 0.81) categories. Lower mean scores were recorded in the psychoticism 1.00 (SD = 0.72) and phobic anxiety 1.02 (SD = 0.84) categories. Confidence interval (CI) parameter indicates the reliability of these estimations (Table 2).

**Table 2:** Mean scores, standard deviation and confidence interval in 9 dimensions of SCL90-R test

Clinical diagnosis	Mean score	Standard Deviation	95% CI	
			
			Lower	Upper
**Somatization**	1.98	0.84	1.840	2.113

**Obsessive-compulsive**	1.51	0.85	1.372	1.652

**Interpersonal sensitivity**	1.37	0.82	1.237	1.506

**Depression**	1.51	0.83	1.372	1.645

**Anxiety**	1.56	0.86	1.417	1.698

**Anger-Hostility**	1.34	0.93	1.189	1.492

**Phobic anxiety**	1.02	0.84	0.882	1.160

**Paranoid ideation**	1.41	0.84	1.267	1.545

**Psychoticism**	1.00	0.72	0.878	1.115

**GSI**	1.46	0.72	1.328	1.588

In all patients, Somatization, obsessive-compulsive, anxiety and depression were the most severe problems with higher mean scores, while psychoticism and phobic anxiety were the least severe problems problems with lower mean scores.

GSI = Global Severity Index.

CI = Confidence interval.

### Demographic and psychological characteristics

The results of this study showed that in participants who had lower education levels, the mean of total GSI scores (effect size = 0.507), somatization (effect size = 0.475), obsessive-compulsive (effect size = 0.519), interpersonal sensitivity (effect size = 0.493), depression (effect size = 0.608), anxiety (effect size = 0.582), anger hostility (effect size = 0.356), and phobic anxiety (effect size = 0.445) were higher versus those with higher education levels (Table 3).

**Table 3:** Association between 9 dimensions of psychological status with demographic characteristics (n = 149)

Items/Status	1	2	3	4	5	6	7	8	9	Total
	
	M (SD)	M (SD)	M (SD)	M (SD)	M (SD)	M (SD)	M (SD)	M (SD)	M (SD)	M (SD)
**Education**										

**< 12 years**	2.18	1.75	1.58	1.76	1.82	1.51	1.22	1.42	1.12	1.66
	(1.80)	(1.78)	(0.81)	(0.77)	(0.80)	(0.95)	(0.87)	(0.82)	(0.69)	(0.64

**≥ 12 years**	1.80	1.32	1.19	1.28	1.34	1.19	0.85	1.39	0.89	1.31
	(0.82)	(1.86)	(0.79)	(0.81)	(0.85)	(0.89)	(0.79)	(0.87)	(0.73	(0.74)

**P-value**	0.005	0.003	0.004	0.004	0.001	0.034	0.009	0.826	0.066	0.007

**Cohen's d**	0.475	0.519	0.493	0.608	0.582	0.356	0.445	0.037	0.312	0.507

**Children(No.)**										

**1-2**	1.72	1.16	1.10	1.26	1.25	1.01	0.72	1.26	0.80	1.21
	(0.76)	(0.76)	(0.75)	(0.82)	(0.82)	(0.84	(0.71)	(0. 84)	(0.71)	(0.67)

**3-5**	2.06	1.67	1.48	1.62	1.66	1.52	1.15	1.53	1.10	1.59
	(0.83)	(0.86)	(0.79)	(0.82)	(0.81)	(0.93)	(0.81)	(0.82)	(0.69)	(0.72)

**> 6**	2.25	1.80	1.53	1.66	1.85	1.44	1.20	1.35	1.10	1.57
	(0.87)	(0.77)	(0.94)	(0.84)	(0.94)	(0.96)	(10.0)	(0.90)	(0.77)	(0.72)

**P-value**	0.018	0.001	0.024	0.040	0.006	0.010	0.010	0. 210	0.062	0.023

**Cohen's d**	0.055	0.102	0.052	0.058	0.072	0.067	0.062	0.016	0.040	0.062

**Employment**										

**Employed**	1.87	1.40	1.13	1.31	1.44	1.25	0.83	1.31	0.85	1.25
	(0.85)	(0.90)	(0.82)	(0.82)	(0.93)	(0.89)	(0.76)	(0.86)	(0.69)	(0.72)

**Unemployed**	2.04	1.59	1.53	1.63	1.64	1.39	1.14	1.47	1.09	1.58
	(082)	(0.81)	(0.79)	(0.82)	(0.80)	(0.95)	(0.86)	(0.83)	(0.73)	(0.69)

**P-value**	0. 230	0.193	0.004	0.023	0.170	0.370	0.029	0.280	0.044	0.017

**Cohen's d**	0.205	0.226	0.501	0.427	0.234	0.152	0.382	0.184	0.348	0.464

**Exposures**										

**Once**	2.01	1.62	1.44	1.59	1.64	1.38	1.10	1.43	1.07	1.52
	(0.89)	(0.90)	(0.88)	(0.87)	(0.87)	(0.99)	(0.91)	(0. 86)	(0.74)	(0.76)

**Twice**	2.02	1.26	1.5	1.39	1.40	1.27	0.83	1.39	0.85	1.32
	(0.75)	(0.75)	(0.72)	(0.78)	(0.86)	(0.81)	(0.66)	(0.90)	(0.72)	(0.65)

**More**	1.63	1.35	1.17	1.26	1.39	1.25	0.92	1.27	0.81	1.33
	(0.56)	(0.56)	(0.57)	(0.64)	(0.68)	(0.78)	(0.68)	(0.65)	(0.53)	(0.50)

**P-value**	0.293	0.082	0.310	0.249	0.263	0.788	0.232	0.081	0.184	0.389

**Cohen's d**	0.017	0.035	0.016	0.015	0.018	0.003	0.021	0.003	0.024	0.016

**Co-morbidity**										

**Yes**	2.01	1.54	1.29	1.49	1.58	1.43	0.92	1.40	0.96	1.45
	(0.82)	(0.83)	(0.73)	(0.78)	(0.84)	(0.89)	(0.80)	(0.86)	(0.70)	(0.69)

**No**	1.94	1.48	1.45	1.52	1.53	1.25	1.11	1.41	1.03	1.47
	(0.85)	(0.87)	(0.90)	(0.89)	(0.88)	(0.96)	(0.88)	(0.84)	(0.75)	(0.74)
**P-value**	0.606	0.685	0.261	0. 815	0.722	0.226	0.174	0.935	0.542	0.898

**Cohen's d**	-0.09	-0.07	0.188	0.139	-0.06	-0.2	0.229	0.014	0.103	0.024

1) Somatization, 2) obsessive-compulsive, 3) interpersonal sensitivity, 4) depression, 5) anxiety, 6) anger-hostility, 7) phobic anxiety, 8) paranoid ideation, and 9) psychoticism

M = Mean GSI, SD = Standard deviation.

In participant who had more than 2 children, the mean of total GSI scores (effect size = 0.062), somatization (effect size = 0.055), obsessive-compulsive (effect size = 0.102), interpersonal sensitivity (effect size = 0.502), depression (effect size = 0.058), anxiety (effect size = 0.702), anger hostility (effect size = 0.607), and phobic anxiety (effect size = 0.062) were higher versus those with less than 2 children (Table 3).

In unemployed participants, the mean of total GSI scores (effect size = 0.464), interpersonal sensitivity (effect size = 0.501), depression (effect size = 0.427), phobic anxiety (effect size = 0.382), and psychoticism scores (effect size = 0.348), were higher versus the employed participants (Table 3).

In patients who had experienced co- morbidity or had experienced more than one exposure to SM, the mean of total GSI scores and all other 9 dimensions of psychological status were not higher versus those without co-morbidity or less frequent exposure to SM (Table 3).

The mean of total GSI scores and all other 9 dimensions of psychological statuses, total PSDI and total PST were higher in participants with positive psychological history versus those with negative psychological history. Confidence Interval (CI) parameter indicates the reliability of these estimations (Table 4).

**Table 4:** GSI mean scores and confidence interval in 9 dimensions of SCL90-R test, based on the psychological history

Psychological dimensions	Positive psychological		Negative psychological		Mean Difference	SD	95%CI	
				
	Mean	SD	Mean	SD			Lower	Upper
**Depression**	1.85	0.79	1.05	0.65	-0.790	0.124	0.543	1.034

**Somatization**	2.23	0.74	1.62	0.83	-0.602	0.131	0.343	0.861

**Obsessive-** **compulsive**	1.81	0.81	1.09	0.72	-0.716	0.132	0.455	0.976

**Interpersonal sensitivity**	1.62	0.82	1.03	0.69	-0.589	0.129	0.335	0.844

**Anxiety**	1.86	0.81	1.13	0.74	-0.734	0.131	0.475	0.994

**Hostility**	1.62	0.91	0.94	0.81	-0.684	0.145	0.397	0.971

**Phobia**	1.26	0.89	0.69	0.64	-0.562	0.135	0.295	0.829

**Paranoid** **indentation**	1.62	0.92	1.11	0.63	-0.515	0.137	0.245	0.786

**Psychoticism**	1.25	0.73	0.66	0.57	-0.586	0.111	0.366	0.806

**GSI total**	1.73	0.68	1.11	0.61	-0.621	0.120	0.384	0.859

**PST total**	64.5	17.49	49.92	20.8	-14.62	3.499	7.693	21.549

**PSDI**	2.36	0.59	1.92	0.54	-0.437	0.104	0.231	0.644

GSI = Global Severity Index

PST = Positive Symptom Total.

PSDI = Positive Symptom Distress Index.

CI = Confidence interval

The higher mean score indicates worst psychological health status in that item. In patients with positive psychological history, the highest mean scores were for somatization, obsessive-compulsive, anxiety and depression. The least mean scores were for phobic anxiety and psychoticism. In patients with negative psychological history, the highest mean scores were for somatization, paranoid ideation and aggression. The least mean score was for phobic anxiety.

## Discussion

The mean GSI in survivors of chemical warfare with ophthalmologic complications was 1.46 (SD = 0.72), which was higher compared to standardized cut-off point (0.4) for Iranian community [24,25]. Based on the SCL-90, the greatest psychological problems were in the categories of somatization, obsessive-compulsive, anxiety and depression.

Since the eyes play an important role in normal function, vision-related psychological health status is an important area that needs to be further understood [27,28]. Given the constant fear that SM survivors have of SM induced blindness, any instability in their psychological status is not surprising [29].

GSI scores were reported by Derogatis in 1002 psychiatric outpatients and 310 psychiatric inpatients and 719 non-patients as 1.32 ± 0.72, 1.36 ± 0.86 and 0.3 ± 0.31 respectively [22]. Similarly, the GSI score of those in our studied population with a positive psychological history was higher compared to cases with negative psychological history.

Neiria et al. reported a mean GSI level of 0.45 in the veterans' cases in contrast with 0.33 in the controls [30]. In another study by Schnurr et al (1996) that looked at individuals exposed to SM during World War II, GSI score was 0.62 [31]. All these scores are lower than the present study. The better scores may be due to better health care services in the countries where the studies were performed or the longer duration between the time of exposure and the study.

In gulf war veterans, PTSD and hospitalization for depression were reported significantly more by deployed troops stationed closer to the explosion site of chemical agents than by non-deployed troops that were farther [32]. The present study revealed that GSI score were worst in those who had a positive psychological history. Torben Ishoy et al. reported a significant correlation between GSI scores and participation in the Gulf War conflict, especially in the categories of obsessive-compulsion and depression, but not for phobic anxiety, paranoid ideation, and psychoticism [33]. In survivors of the Kosovo war, being a refugee was associated with a higher likelihood of having social anxiety disorder, and major depressive disorder [34]. According to the study done by Bramsen et al, higher suicidal thoughts, and depression were associated with GSI scores above 1 in World War II survivors [35]. In the present study, the highest mean scores were in the categories of somatization, anxiety, and depression and the lowest ones were in the categories of psychoticism and phobic anxiety.

Mousavi et al. reported the mean GSI scores of war-related bilateral lower limb amputees to be 0.88. The scores in all 9 dimensions of SCL-90 were lower than the present study [36]. Therefore, the worse GSI scores in this survey signify poorer psychological health status in chemical warfare survivors with ophthalmologic complications. This might be due to the progressive nature of the chemical agents induced injuries, compared to constant nature of the defects on extremities [29].

Based on the findings of this study, and considering effect of sample size (Cohen's d), there were better psychological health status and lower GSI scores in patients who were employed and had higher levels of education. Aside from SM toxicity, these findings seem logical and no explanations are needed. There were poor psychological health status and higher GSI scores in patients who had higher number of children. Of course lower Cohen's d indicates a necessity of larger sample size in this regard. In the case of the impact of higher number of children on worsening of psychological health status, in addition to SM effects, this factor may influenced on subsistence and economical living of each family head and more fear from inability to manage the living expenses with higher numbers of children. There were poor psychological health status and higher GSI scores in patients who had a history of psychological disorder. For evaluating of SM impacts on worsening of psychological health status after exposure one may needs the previous psychological data of same types that lacked in this study. So the actual relevance of exposure and worsening of psychological symptoms should be outlined cautiously, especially without having any familial psychological history that we were encountered as a limitation in this study. The results of this study revealed that the mean of total GSI scores and all other 9 dimensions of psychological status, were not higher in patients who had experienced co- morbidity. This may be due to the more important effects of vision on living independently in contrast to the other comorbidities. In addition there were no worst psychological health statuses in total GSI scores and in all other 9 dimensions, in patients who had experienced more than one exposure to SM. This finding suggests that most of the fears may come from the first exposure. However, this is in contrast to a study of 24 men who volunteered to participate in sulfur mustard chamber tests. In that study, the number of exposures to SM was the only factor that predicted lifetime full or sub-diagnostic forms of PTSD [31]. Co-morbidity was not associated with higher GSI scores. This finding might reflect the importance of the ocular injuries and its impact on the health of survivors in comparison with damage to other organs. On the other hand these unexpected findings in recent two items may be due to the effect of sample size (lower Cohen's d).

The strength of this study is its ability to uncover the individual and social factors affecting mental health status in soldiers with exposure to chemical munitions. The weakness of this study was the lack of a control group. Also this experience was a descriptive study and we could not find significant association between different variables possibly due to small effect size. Supplementary researches with controls and/or greater sample size may be helpful for any further conclusion.

The findings of this study confirm the destructive effects of chemical warfare agents on the psychological health status of the victims. The results especially emphasize the possibility that SM induced ocular injury can trigger changes in psychological health status for decades. Based on the associations found in this study, increasing educational levels, and bearing fewer children and creation of appropriate jobs may provide a more sweet life by reducing mental stress in these patients.

## Conclusion

Ocular injuries induced by SM, aside from systemic insult may trigger destructive effects on psychological health status of victims. Identification of this high risk population and providing appropriate set-up and suitable educations may help to improve psychological health status in these patients.

## Abbreviations

GHQ: General Health Questionnaire; GSI: Global severity index; GW: Gulf War; JMERC: Janbazan Medical and Engineering Research Center; PSDI: Positive Symptom Distress Index; PST: Positive Symptom Total; PTSD: Post-Traumatic Stress Disorder; SCL-90-R: Symptom Check List 90-Revised; SM: Sulfur mustard.

## Competing interests

The authors declare that they have no competing interests.

## Authors' contributions

Gh G: Substantial contributions to conception and design; HG: Final approval of the version to be published and corresponding author; BM: Revising the manuscript critically for important intellectual content; MR S: Conceived of the study, and participated in its design and coordination; PR: Interpretation of data; FJ design surveys and experiments; MMN: Analysis and interpretation of data; SAM: Study design and patients evaluation; M SN: Acquisition of data and. involved in drafting the manuscript. All authors read and approved the final manuscript.
